# *Staphylococcus aureus* Toxins and Their Molecular Activity in Infectious Diseases

**DOI:** 10.3390/toxins10060252

**Published:** 2018-06-19

**Authors:** Diana Oliveira, Anabela Borges, Manuel Simões

**Affiliations:** LEPABE, Department of Chemical Engineering, Faculty of Engineering, University of Porto, Rua Dr. Roberto Frias, s/n, 4200-465 Porto, Portugal; dianarosalopesoliveira@gmail.com (D.O.); apborges@utad.pt (A.B.)

**Keywords:** biofilms, epidemiology, exfoliative toxins, pore-forming toxins, *Staphylococcus aureus*, superantigens

## Abstract

*Staphylococcus aureus* is a microorganism resident in the skin and nasal membranes with a dreadful pathogenic potential to cause a variety of community and hospital-acquired infections. The frequency of these infections is increasing and their treatment is becoming more difficult. The ability of *S. aureus* to form biofilms and the emergence of multidrug-resistant strains are the main reasons determining the challenge in dealing with these infections. *S. aureus'* infectious capacity and its success as a pathogen is related to the expression of virulence factors, among which the production of a wide variety of toxins is highlighted. For this reason, a better understanding of *S. aureus* toxins is needed to enable the development of new strategies to reduce their production and consequently improve therapeutic approaches. This review focuses on understanding the toxin-based pathogenesis of *S. aureus* and their role on infectious diseases.

## 1. Introduction

*S. aureus* is an opportunistic human pathogen known to colonize the respiratory tract of approximately 30% of the U.S. population [1,2]. *S. aureus* infections range in severity from mild skin infections to severe necrotizing pneumonia. It is simultaneously the leading cause of bacteremia, infective endocarditis (IE), and can also cause osteoarticular, skin and soft tissue, pleuropulmonary, and device-related infections [3]. Vancomycin is commonly used to treat these infections as this antibiotic is active against both methicillin-resistant and methicillin-susceptible *S. aureus* strains (MRSA and MSSA). However, over the years the emergence of resistant strains to antimicrobials has been reported, the main reason why these infections are becoming more difficult to treat [4,5].

*S. aureus* can express a variety of resistance mechanisms and virulence factors, allowing it to evade host natural defenses [6]. Among the multiple virulence factors produced by this microorganism, it is possible to enumerate toxins, enzymes and cell surface-associated antigens [6]. Toxins can lead to a weak response by the colonized organism since they are able to degrade some of the host cells, manipulate the innate and adaptative immune responses, and degrade inter-cellular junctions, which has an obvious contribution in *S. aureus* proliferation [7].

Thus, a strong correlation between virulence genes and certain disease symptoms has been established. For instance, toxins are being strongly suspected to cause diseases such as toxic shock syndrome (TSS), staphylococcal scalded skin syndrome (SSSS), necrotizing pneumonia, or deep-seated infections [8,9,10,11,12]. This clear association between toxins and some diseases has led to an urgent and better understanding of *S. aureus* pathogenesis, etiology, epidemiology, pathophysiology and more precisely its high regulated mechanism of toxin production. Effective preventive approaches will enhance the capacity to control staphylococcal infections, as the tenacity of this microorganism as a pathogen is mainly attributed to its machinery of virulence factors [13,14].

## 2. *Staphylococcus aureus* Etiology, Epidemiology and Pathophysiology

*S. aureus* is a major pathogen that causes a wide variety of infectious diseases [3]. This bacterium has a cell wall composed of a single lipid membrane, surrounded by a thick layer of peptidoglycan and lipoteichoic acid anchored by diacylglycerol [15]. Peptidoglycan is the main compound of the staphylococcal cell wall (50%) and consists of two alternating polysaccharide subunits of *N*-acetylglucosamine and *N*-acetylmuramic acid with 1,4-β linkages [16]. The peptidoglycan chains give rigidity to the cell wall, determining its shape and protecting it from osmotic lysis [16]. Another cell wall constituent is a group of phosphate-containing polymers called teichoic acids, which contribute for 40% of the cell wall mass [17]. Teichoic acids give a negative charge to the staphylococcal cell surface and play a role in the acquisition and localization of metal ions [18]. Together, these two cell wall components account for 90% of the weight, the remaining 10% being composed of surface proteins, exoproteins and autolysins [19].

*S. aureus* was first identified in 1884 by Anton Rosenbach, a German surgeon [20,21]. Rosenbach isolated two strains of staphylococci (*S. aureus* and *Staphylococcus epidermidis*) and named them based on the pigmented appearance of their colonies [20]. At the time of this discovery, most of the patients infected with *S. aureus* died, with a mortality rate of near 82% for patients with *S. aureus* bacteremia [22]. This rate largely decreased after the introduction of penicillin into clinical practice [23]. However, in the early 1940s, the first penicillin-resistant *S. aureus* strains appeared and by the end of the decade, almost 25% of the hospital-associated strains were resistant to penicillin [24,25,26]. Though methicillin and oxacillin were developed to overcome this resistance problem in the 1960s, only one year after their introduction, resistant strains emerged again, designated as MRSA [27]. Therefore, between the 1960s and the 1980s, MRSA hospital-acquired infections become more frequent, and in the 1990s the percentage of infections related to MRSA reached 29% [28,29]. Since then, it has been shown that contact with patients with MRSA and the receipt of antimicrobials increased the probability of being infected with this microorganism [26,30,31]. However, in the late 1990s, MRSA infections started to be reported in healthy, young patients who had no exposure to a hospital setting [32,33,34,35]. Today, hospitalized patients are still the most predisposed to acquire these infections mainly because of their compromised immune system, extended exposure to antimicrobials, and the use of contaminated indwelling devices such as ventilators or catheters [36,37]. 

Therapeutic options available are typically ineffective due to the strong ability of *S. aureus* strains to develop antibiotic resistance determinants [38]. Thus, the control of bacterial infections requires a deep understanding of intracellular genetics and biochemistry, and profound knowledge on how the biofilm mode of life affects antibiotic action and the development of resistance [38]. Once bacteria are immersed in biofilm, they have community resistance mechanisms distinct from the well-characterized single cell level mechanisms [38]. Bacterial communities exhibit an altered phenotype in terms of growth, gene expression and protein production, and can also exhibit tolerance to environmental stress that single cells cannot [39,40]. The matrix of polymeric substances (EPS) that surrounds these bacteria protects from phagocytosis, antibiotics, disinfectants and other components of the innate and adaptive immune and inflammatory systems of the host, and allows the capture of nutrients such as carbon, nitrogen and phosphate, which are essential for the cell [39,41,42]. 

The multi-layered biofilm produced by *S. aureus* is embedded within an EPS matrix, which is described to be mainly composed of teichoic acids (80%) and staphylococcal and host proteins [43]. A more detailed EPS characterization identified a polysaccharide intercellular antigen (PIA), also known as polymeric *N*-acetylglucosamine (PNAG) [44]. PIA is composed of β-1,6-linked *N*-acetylglucosamine residues (80–85%) and an anionic fraction with a lower content of non-*N*-acetylated d-glucosaminyl residues that contains phosphate and ester-linked succinate (15–20%) [45].

As part of the natural biofilm development cycle, cells within mature biofilms produce compounds that can induce shift from biofilm to a planktonic mode of life [38]. This shift is essential in the process of cell dispersal from biofilms [46]. In *S. aureus*, biofilm detachment is controlled by the quorum-sensing system Agr [47,48]. This system is suggested to be an important regulatory switch between planktonic and biofilm regimes, contributing to dispersal and colonization of *S. aureus* in other sites [48]. *S. aureus* quorum-sensing system is encoded by the accessory gene regulator (agr) locus, and it produces a communication molecule called an autoinducing peptide (AIP) [49]. Once AIP reaches a critical concentration, a regulatory cascade is initiated, and a myriad of virulence factors is expressed. This system consists of two divergent transcripts, RNAII and RNAIII, initiated from two distinct promoters, P2 and P3, respectively. The P2 operon contains agrBDCA and codes for the RNAII transcript, while P3 drives transcription of the effector molecule of the agr locus (RNAIII) [47,49]. An increase in the transcription of P2 and P3 appears to result in a rise of the intracellular concentrations of RNAIII, which in turn, seems also to increase the expression of secreted virulence factors [47]. 

Many of the secreted *S. aureus* virulence factors can be described as toxins, as they are usually described as poisonous substances [50]. On the other hand, toxins are distinct from other virulence factors as they are secreted by the producing organism and they directly affect the host [50]. The influence of some toxins in biofilm development has been studied and it has been demonstrated that some of them play a preeminent role in biofilm progress. For example, Scheer et al. demonstrated the influence of Hemolysin-α (Hla or α-toxin) and Leukotoxin AB (LukAB) in biofilm persistence [51]. The importance of these toxins was assessed using a murine orthopaedic implant biofilm infection model and revealed a synergistic role of Hla and LukAB in promoting macrophage dysfunction and eliciting cell death. This reduction in macrophage phagocytosis facilitated the ability of *S. aureus* to avoid destructive host responses when organized as a biofilm [51]. Another study demonstrating the influence of toxins in biofilm progression was conducted by Dastgheyb showing that phenol-soluble modulins (PSMs) interfere with biofilm formation by disrupting interactions of matrix molecules with the bacterial cell surface [52]. The interaction of *S. aureus* surface proteins with host matrix proteins such as fibrin initiates agglomeration and it grows to exceptionally large dimensions due to the lack of PSM expression under the specific conditions of the study [52]. An in vitro study conducted by Periasamy demonstrated similar results with regard to the impact of *S. aureus* PSMs in biofilm formation, as PSMs destructed biofilms and led to its dispersal due to the surfactant properties of the toxin [53].

## 3. *Staphylococcus aureus* Toxins

The proficiency of *S. aureus* as a pathogen can be attributed to its arsenal of virulence factors among which secreted toxins play an important role [13,14]. The main *S. aureus* toxins can be divided into three major groups—the pore-forming toxins (PFTs), exfoliative toxins (ETs) and superantigens (SAgs). Pore-forming toxins can be further divided into four types—Hemolysin-α (Hla or α-toxin), Hemolysin-β, leukotoxins and phenol-soluble modulins (PSMs) [7]. This system of protein production responds to a wide range of different conditions and understanding this mechanism will allow a better control of staphylococcal infections. 

*S. aureus* toxins are related to some diseases such as toxic shock syndrome (TSS), staphylococcal scalded skin syndrome (SSSS), necrotizing pneumonia or deep-seated skin infections [8,9,10,11,12]. The toxins are capable of damaging the cells membranes of the host, either by degrading inter-cellular connections or by modulating immune responses [7].

### 3.1. Pore-Forming Toxins

#### 3.1.1. Hemolysin-α (Hla or α-Toxin)

**Figure 1:**
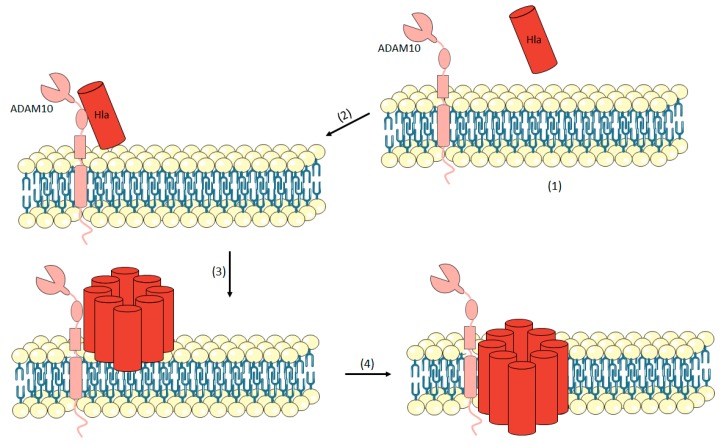
Mechanism of action of Hemolysin-α (Hla). Hla is secreted as a water-soluble monomer (1). Hla binds to the transmembrane protein ADAM10 which is a cellular receptor for α-toxin (2). Then, the toxin oligomerizes into a heptamer on the plasma membrane and a pre-pore is formed (3) and, at the end, the formation of the transmembrane channel occurs (4).

Hla is a 33 kDa polypeptide secreted by 95% of clinical *S. aureus* strains [6,7]. Hla is a beta-barrel forming toxins secreted as a water-soluble monomer [7,50]. This toxin on its own is not considered toxic, it is its bonding capability and oligomerization into a heptameric structure on the host cell membrane that makes it dangerous [54,55]. When Hla binds to its target cell, it oligomerizes to a pre-pore structure and attacks the cell membrane by extrusion of the β-barrel through the lipid bilayer to form a hydrophilic transmembrane channel (Figure 1) [56]. This toxin is the prototype for the class of small β-barrel pore-forming cytotoxins (PFTs) [57,58,59,60]. Over the years, pore formation and cellular lysis were considered the most prominent consequence of Hla action. However, recent studies have shown the importance of the alteration of the cell signalling pathways, which includes cell proliferation, inflammatory responses, cytokine secretion and cell-cell interactions [54,55,57,58,59,60,61]. This toxin has been demonstrated to affect a wide range of human cell types, including epithelial cells, endothelial cells, T cells, monocytes, and macrophages [57,62,63,64,65,66,67,68].

Hla induces different signalling events in the target cell, depending not only on the relative concentration of toxin but also on the type of cell exposed [64]. When the toxin approaches the cell, the small pore formed allows rapid release of ATP, *K^+^* ions, while also restricting the movement of macromolecules across the cell membrane [64,69,70,71]. After the pore formation step, there is an influx of extracellular calcium into the cell. The increase in the intracellular calcium stimulates hydrolysis of membrane phospholipids and metabolism of arachadonic acid to leukotrienes, prostanoids and thromboxane A2 [72,73,74]. Then, there is also an activation of the protein Kinase C and the induction of NF-κB nuclear translocation [72,74,75]. All these events together with the production of IL-1β, IL-6 and IL-8 signify the pro-inflammatory stimulus. In the epithelial cells, which are key targets of Hla, E-cadherin is a main substrate for ADAM10 [76,77]. ADAM10, a cellular receptor for Hla, is a zinc-dependent metalloprotease expressed as a type I transmembrane protein on the surface of most host cells [78,79]. The enzyme that degrades E-cadherin is activated after Hla bond compromising the epithelial tissue barrier function which in turn allows *S. aureus* invasion [7,80]. The discovery of ADAM10 as a cellular receptor for Hla allows a deeper examination on the effects of the toxin in a specific cell population. It has been verified that this toxin plays an important role in *S. aureus* pathogenesis and there are two evidences reinforcing this hypothesis. The first concerns people who suffer from *S. aureus* disease and develop serum antibody responses to the toxin consistent with the toxin expression [81,82,83]. The other evidence is regarding bacterial genetic and protein profiling analysis [64]. From analysis of the hla and agr loci of the *S. aureus* strains involved in epidemic events in the 1950s and 1960s, it was possible to reveal the capability for Hla expression, confirmed by the presence of a high virulent phenotype of these isolates in animal studies of Hla-mediated disease [84]. Together these studies show that the expression of Hla may be required for the pathogenesis of invasive disease in healthy individuals [64]. 

#### 3.1.2. Hemolysin-β (Sphingomyelinase C)

Hemolysin-β was firstly identified in 1935 by Glenny and Stevens and the sequence of the gene *hlb* was discovered in 1989 by Projan et al. [85,86]. This toxin has shown to be highly hemolytic towards erythrocytes in sheep, but not in rabbits [8]. The difference in susceptibility for erythrocytes may be due to the different sphingomyelin contents of these cells since the toxin is also known as sphingomyelinase [87]. Sphingomyelinases are phosphoric diester hydrolases that cleave sphingomyelins, the most abundant sphingolipid in eukaryotic membrane [88]. 

The role of this toxin in disease is not clearly understood. It is known that β-hemolysin is produced in large amounts in animal isolates and its high expression may indicate that β-hemolysin producers have some selective advantage from toxin secretion [8]. This toxin is produced in large quantities in strains isolated from bovine mastitis [89] and human skin chronic infections [90]. β-hemolysin is cytotoxic towards human keratinocytes, polymorphonuclear leukocytes, monocytes, and T lymphocytes and inhibits interleukin-8 (IL-8) expression by endothelial cells. These can contribute to phagosomal escape of *S. aureus* and induction of biofilm formation [90,91,92,93,94]. 

Some studies have demonstrated the importance of this toxin for *S. aureus* pathogenicity. Virulence of a mutant *S. aureus* strain lacking hlb was reduced in pneumonia and murine ear skin infection models [90,95]. In addition, a mutant strain expressing a biofilm formation-deficient β-hemolysin had a reduced pathogenicity in an endocarditis model in rabbits [92].

#### 3.1.3. Leukotoxin

The pore-forming leukotoxins consist of two different protein components that assemble together to form β-barrel pores [96,97]. So far, it has been isolated from *S. aureus* strains associated to human infections four bi-component leukotoxins that are structurally similar to Hla: Panton-Valentine Leukocidin (PVL), gamma (γ)-Hemolysin (HlgA, HlgC, HlgB), Leukotoxin ED (LukE, LukD) and Leukotoxin AB/GH (LukAB/LukGH) [98]. These pore-forming toxins consist of two different protein components classified as “S” and “F”. The S-component confers cell-type specificity by binding to cellular receptors (Table 1) [96,97,98].

**Figure 2:**
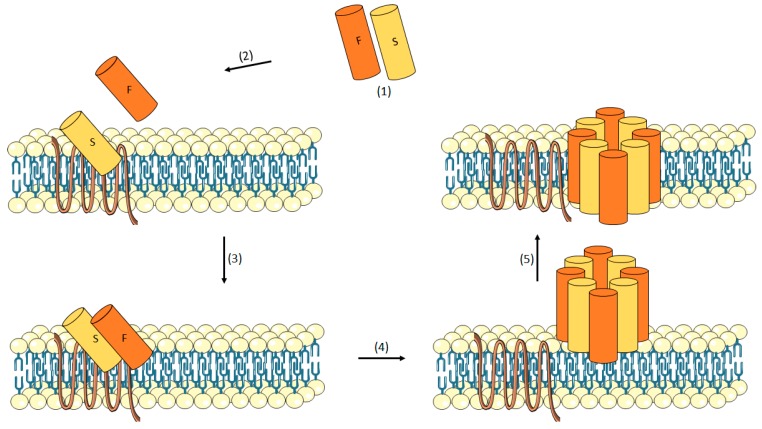
Mechanism of action of leukotoxins. The monomers are secreted (1). The S-component binds the cell surface receptor (2) then, the F component is recruited, and dimerization occurs (3). These dimers oligomerize on the plasma membrane and a pre-pore appears (4). At last, the formation of the transmembrane channel occurs (5).

After bonding, a conformational change allowing dimerization with F-components is induced [99]. Then, an oligomerization occurs to form the pre-pore prior to insertion of the β-barrel transmembrane channel [100]. There is strong evidence corroborating this hypothetical mechanism [101,102,103]. Miles et al. described the formation of an octameric pore containing four subunits each of LukF and LukS [101]. Further, images of purified γ-Hemolysin pores on human erythrocyte membranes obtained by electron microscopy were interpreted as heptamers [102]. Additionally, Jayasinghe et al. provided convincing evidences that the Luk pore is an octamer formed by LukF and LukS and assembled on rabbit red cell membranes [103]. These authors also show the existence of four LukF and four LukS subunits [103]. The mechanism of pore formation of these bi-component toxins with the membrane of the cell is shown in Figure 2. 

The four leukotoxins lyse cells of the leukotocytic lineage and they also have been known to kill neutrophils, while only γ-Hemolysin and LukED have demonstrated lytic activity against red blood cells [98,104,105]. 

##### γ-Hemolysin (HlgAB/HlgCB)

Within the γ-Hemolysin group, HlgAB, HlgCB or HlgACB are three proteins with different subunit combinations. HlgAB and HlgCB share the same F subunit (HlgB) but differ in S subunit composition (HlgA or HlgC) [98,106]. HlgA (32 kDa) and HlgC (32k Da) only exhibit lytic activity when combined with HlgB (36 kDa) [107]. HlgAB is particularly effective at lysing human red blood cells and exhibits cytolytic activity towards human and rabbit leukocytes [104,108,109]. In contrast, HlgCB exhibits limited activity towards red blood cells [56]. The role of HlgACB in *S. aureus* virulence is not well understood. It is only known that *hlgACB* locus is quite associated with strains able to cause human and bovine colonization, but no association with a particular infection type has been found yet [110,111,112].

HlgAB was described in 1938 but only in the late 1970s was its biological and biochemical functions assigned due to more reliable purification methods [113,114]. Today, HlgAB has been shown to be required for *S. aureus* survival and proliferation during bloodstream infection, likely through macrophage evasion and nutrient (Fe^2+^) release from erythrocytes [108,115].

##### LukED

LukED was initially reported over a decade ago. However, research about this leukotoxin has remained minimal [105,116]. LukED has shown its cytolytic activity against rabbit blood cells and leukocytes, but subsequent studies show that this toxin is the only one capable of killing mouse phagocytes with high efficiency [56,117,118]. According to epidemiological data, although this toxin is widespread, it is prevalent in epidemic strains [56]. LukED genes have been isolated in 87–93% of the clinical strains in Japan and 88–99% in MRSA strains around the world, suggesting the potential contribution of this toxin for *S. aureus* pathogenicity [98,105,119,120].

##### LukAB/GH

LukAB (also known as LukGH) is the most recent toxin discovered within the leukotoxin group and the role of this toxin during in vivo infection still remains unclear [121,122]. On the other hand, in vitro studies reported that LukAB/GH targets monocytes, dendritic cells and leukocytes and can cooperate with Panton-Valentine leucocidin (PVL) when co-expressed [110,122,123]. This bi-component leukotoxin is the only one known to enhance *S. aureus* survival as it plays a role in escaping from phagocytes and neutrophils [122,124]. The mechanism under which this toxin promotes survival of *S. aureus* is likely to be related to its toxicity towards neutrophils, as no hemolytic activity is detectable in the whole blood [122]. Recently, the contribution of LukAB/GH to virulence was demonstrated using a systemic infection model, where the presence of the toxin was associated with higher bacterial burden in infected kidneys [122].

Unlike other leukotoxins, which are secreted as monomers that oligomerize after binding to the cell surface, LukAB/GH heterodimerize before binding its cell-surface receptor. This suggests that LukAB/GH dimerization occurs prior to secretion, as the co-expression of subunits appear to be more toxic than the mixture of individually purified subunits [125,126]. 

##### Panton-Valentine Leucocidin (PVL)

Panton-Valentine Leukocidin (PVL) was firstly purified by Woodin from the culture supernatants of *S. aureus* V8 [127]. From this purification step, it was possible to separate two protein components of 32 kDa and 38 kDa referred as S, for slow-eluted, and F, for fast-eluted, based on their migration on carboxymethyl cellulose column [127]. All PVL-producing isolates produce the class S and class F proteins specific for PVL (LukS-PV and LukF-PV) and all PVL genes (lukS-PV and lukF-PV) are encoded in several bacteriophages expressing Sa2 integrase [128,129,130].

The contribution of this leukotoxin in *S. aureus* virulence is not yet conclusively proven [98]. It is known that PVL is present in a small percentage (approximately 5%) in clinical *S. aureus* strains, but it is strongly associated with community acquired MRSA strains (approximately 85%), particularly those causing pneumonia, and skin and soft tissue infections [98]. This linkage to virulent strains suggests its capability of causing deadly infections in healthy people [131,132]. A clinical study has shown that pneumonia associated to PVL-positive *S. aureus* was more lethal compared to PVL-negative [133]. Autopsy of these patients revealed that those with PVL-positive *S. aureus* had ulcerated and hemorrhagic lungs, suggesting extreme inflammation. In fact, the PVL-induced lysis of macrophages and neutrophils was attributed to the severe inflammation [133,134].

PVL is a cytotoxin that affects leukocytes and causes tissue necrosis and has been associated to furuncles, cutaneous abscesses and severe necrotic skin infections [135,136,137]. However, the PVL genes were neither detected in strains responsible for endocarditis, mediastinitis, hospital-acquired pneumonia, urinary tract infections and enterocolitis, nor in toxic-shock syndrome [6]. 

#### 3.1.4. Phenol-Soluble Modulins

Phenol-soluble modulins (PSMs) were discovered in 1999 by Mehlin et al. in a *S. epidermidis* culture [138]. In that study, three peptides were identified—PSMα, PSMβ and PSMγ—and were described as a pro-inflammatory complex isolated by hot phenol extraction from the culture filtrate [138,139,140]. PSMγ is termed also as δ-toxin as it is identical to the *S. epidermidis* δ-toxin [141]. 

Studies also led to the identification of PSMs in *S. aureus* [140]. In *S. aureus*, PSMs are encoded at three different locations in the genome [140]. PSMα peptides (PSMα1-PSMα4) are encoded in the psmα operon; PSMβ (PSMβ1, PSMβ2) are encoded in psmβ operon; and δ-toxin is encoded within the coding sequence for RNAIII [142]. 

PSMs peptides form an α-helix amphipathic structure, which stretches over virtually the entire length of the peptide in the shorter α-type PSMs, and are located in the carboxy-terminal region in the longer β-type PSMs. PSMs are believed to attach to the cytoplasmic membrane in a non-specific way, which in turn, can lead to membrane disintegration [50]. It has been considered that phospholipid composition and membrane charge are important for cell susceptibility to PSMs [50].

**Figure 3:**
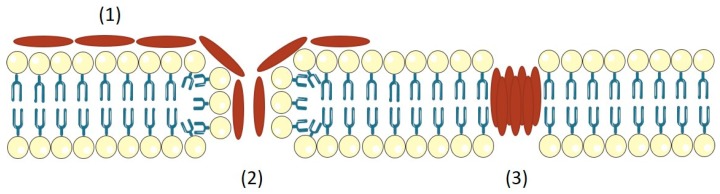
Model of pore-formation mechanism for *S. aureus* PSMs. PSMs attach the cytoplasmic membrane in a non-specific fashion (1), this can lead to membrane disintegration (2). PSMs have the tendency to aggregate in oligomers and form a pore, which is short-lived (3).

PSMs differ in their charge characteristics. PSMα are positively charged and PSMβ are negatively charged, while δ-toxin is neutral [142]. The tendency of PSMs to aggregate in oligomers, forming short-lived pores (Figure 3); and their capacity to facilitate spreading on surfaces or to structure biofilms appears to be the main aspects in pathogenesis [142]. Wang et al. demonstrated that PSMα peptides have a key impact on the ability of community associated MRSA to cause skin infection and bacteraemia; δ-toxin and PSMβ have little or no effect on the mouse models of *S. aureus* infection [140]. 

Globally, PSMs peptides in *S. aureus* have biofilm-structuring activities, indicating their influence on biofilm development via shared physico-chemical properties [53]. Moreover, it is believed that the production of PSMs is highly correlated to the capacity of the staphylococcal species to cause invasive infections, due to their ability to lyse human neutrophils and stimulate inflammatory responses [53,140,143].

### 3.2. Exfoliative Toxins (ETs)

**Figure 4:**
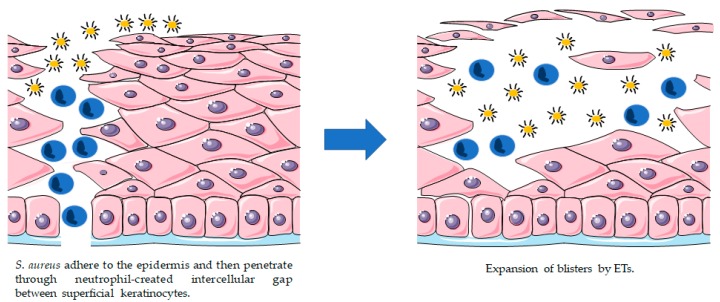
Proposed model for bacterial invasion and blistering provoked by staphylococcal ETs.

Exfoliative toxins (ETs), also known as epidermolytic toxins, are extremely specific serine proteases secreted by *S. aureus*. These proteases recognize and hydrolyse desmosome cadherins in the superficial layers of the skin [144,145]. ETs are exotoxins associated with the cleavage of keratinocytes junctions and cell-cell adhesion in the epidermis of the host, which can induce skin peeling and blister formation (Figure 4) [146,147,148,149]. 

In 1878, von Rittershain described the clinical features of epidermal exfoliation in newborns [150]. However, the relationship between the exfoliation and *S. aureus* was only discovered in 1967 by Lyell [151]. The delay between Ritter’s and Lyell’s discovery was caused by the fact that the blister fluid and exfoliated regions are often free of cultivable staphylococci, because the toxin is distributed from distant sites of infection through the bloodstream [145]. In 1972, Melish et al. discovered the existence of a hypothetical toxin, previously suggested by Lyell, and demonstrated the pathogenic role of those toxins in newborn mice (which were used as experimental models) [152]. 

The principal ETs are known so far are the exfoliative toxin A/B/C/D (ETA, ETB, ETC, ETD). ETA and ETB are the most implicated in human skin damage, while ETC was only isolated from a horse infection and no association with human disease was found [144,153]. ETD was only identified in 2002 in a clinical sample of *S. aureus* [146]. These ETs are produced by approximately 5% of *S. aureus* strains, ETA being most prevalent in Europe, Africa and America and ETB more common in Japan [154]. The production of ETs in certain *S. aureus* strains is related to localized epidermal infections such as bullous impetigo and generalized diseases like SSSS [144]. 

SSSS is a syndrome characterized by skin exfoliation, but its early manifestations include fever, skin hypersensitivity, and erythema, followed by superficial fluid-filled blister formation and skin separation [145,155,156,157,158]. SSSS can affect a large part of the body. However, when a more restricted area is affected, it is categorized as bullous impetigo. Both conditions share the same etiology and only differ in the amount of affected skin [145]. SSSS occurs mainly in infants and children, as they have immature immune systems and weak renal clearance of toxins [12,145]. In general, however, when children are submitted to appropriate treatment, the mortality percentage is quite low (approximately 5%) [159,160]. In contrast, when this syndrome affects adults, especially those with immunosuppressed systems, it has a death rate of approximately 59% [159]. 

### 3.3. Superantigens (SAgs)

Originally, Superantigens (SAgs) were termed as staphylococcal enterotoxins (SEs) because they cause symptoms typical of *S. aureus* food poisoning such as vomiting and diarrhea [7]. However, as some of the most recent identified toxins belonging to this group did not present these emetic properties, in 2004, the International Nomenclature Committee gave SE this new nomenclature [7]. There are more than 23 staphylococcal SAgs toxins described, particularly the toxic shock syndrome toxin (TSST-1) and the staphylococcal enterotoxins (SEA to SEE, SEG to SEJ, SEL to SEQ and SER to SET), and 11 staphylococcal superantigen-like (SSL) toxins (SEIK to SEIQ, SEIU to SEIX) [7,9,161,162,163,164,165]. 

**Figure 5:**
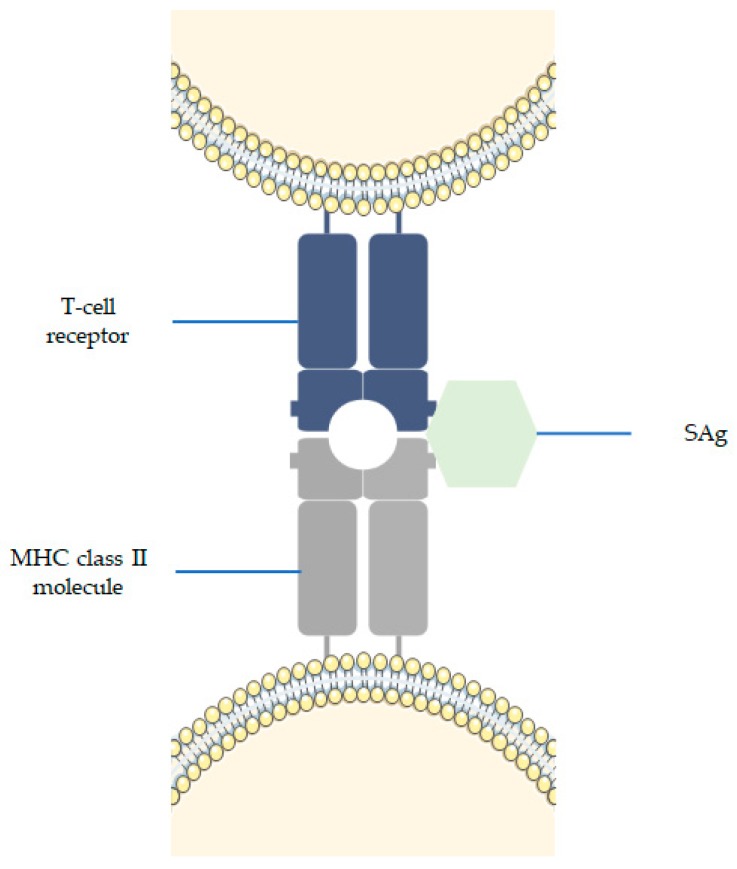
Mechanism of action of SAgs. SAg bind to MHC class II molecules and to a variable region of T-cell receptor, which leads to the stimulation of many T-cells.

The action mechanism of SAgs was first described by Bernhard Fleischer and Hubert Schrezenmeier in 1988. Since then, it is believed that SAgs activate a large fraction of T lymphocytes simultaneously by directly cross-linking certain T cell receptor Vβ domains with conserved structures on major histocompatibility complex class II (MHC II) molecules (Figure 5) [6,161,166,167]. MHC II molecules appear to impact the ability of macrophages to regulate T cell response to SAgs [6]. The released SAgs act systemically, triggering a large number of T-cells to produce massive amounts of pro-inflammatory cytokines (IL-2, IFN-γ and TNF), thereby causing the manifestation of symptoms (e.g., high fever, rash, desquamation, vomiting, diarrhea, hypotension, and frequently can result in multiple organ failure) [168,169]. After this cytokine storm, a lack of response by T-cells where T-cells fail to proliferate or secrete IL-2 is followed, or they can undergo cell death [170,171]. This suggests that SAgs are potent immunogens, eliciting and neutralizing antibody response [161,167]. 

The role of SAgs in some diseases such as sepsis, skin and airway allergies is a matter of discussion [172,173,174,175,176]. For example, from the analysis of patients that suffer from chronic rhinosinusitis, commonly accompanied by intrinsic asthma, it was found that they possess high titres of SAg-specific IgE antibody in the serum or in the polyps, suggesting that SAgs drive or at least amplify chronic airway inflammation [172,175].

## 4. Conclusions

Staphylococci have developed a highly regulated toxin production system that researches are only beginning to understand. However, there is a clear correlation between these toxins and some threatening human diseases. Toxins are attractive key targets for innovative therapeutics. Among the possible strategies, the use of neutralizing antibodies and vaccines are the most promising ones [177,178,179,180]. Vaccines against *S. aureus* should induce antibodies to prevent bacterial adherence and allow the neutralization of the toxic exoproteins produced by the bacterium. However, the development of vaccines will be an extraordinary challenge since there is a large number of toxins available and the production of toxins can vary significantly with the staphylococcal genotype. For this reason, a broad spectrum anti-toxin vaccine containing multiple toxins will be needed. Yet, this therapeutic approach should be customized to each patient and adapted to its natural or induced antibody response. 

Further research on the pathogenesis of staphylococcal infections will enable the design of a more effective *S. aureus* therapeutic as the strategy of simultaneously using a mixture of inhibitors/antibodies targeting multiple toxins is still incipient. 

## Figures and Tables

**Table 1:** Protein subunits “S” and “F” of each leukotoxin and cell specificity [98].

Leukotoxin	S Subunit	F Subunit	Cell Specificity
PVL	LukS-PV	LukF-PV	Leukocytes and neutrophils
Luk AB/Luk GH	LukA/LukH	LukB/LukG	Human monocytes, dendritic cells, neutrophils and leukocytes
LukED	LukE	LukD	Rabbit blood cells and leukocytes, human neutrophils and mouse phagocytes
γ-Hemolysin	HlgA, HlgC	HlgB	Human red blood cells, neutrophils and human and rabbit leukocytes
